# Comparative proteomics of adult *Paragonimus kellicotti* excretion/secretion products released in vitro or present in the lung cyst nodule

**DOI:** 10.1371/journal.pntd.0010679

**Published:** 2022-08-17

**Authors:** Lucia S. Di Maggio, Kurt C. Curtis, Petra Erdmann-Gilmore, Robert S. W. Sprung, R. Reid Townsend, Gary J. Weil, Peter U. Fischer

**Affiliations:** 1 Division of Infectious Diseases, Department of Medicine, Washington University School of Medicine, St. Louis, Missouri, United States of America; 2 Division of Endocrinology, Metabolism and Lipid Research, Department of Medicine, Washington University School of Medicine, St. Louis, Missouri, United States of America; 3 Department of Cell Biology and Physiology, Washington University School of Medicine, St. Louis, Missouri, United States of America; University of Liverpool, UNITED KINGDOM

## Abstract

*Paragonimus kellicotti* is a zoonotic lung fluke infection, the agent of North American paragonimiasis, and an excellent model for other *Paragonimus* infections. The excretory/secretory proteins (ESP) released by parasites and presented at the parasite-host interface are frequently proposed to be useful targets for drugs and/or vaccines In vitro culture conditions may alter ESP compared to those produced in vivo. In order to investigate ESPs produced in vivo we took advantage of the fact that adult *P*. *kellicotti* reproduce in the lungs of experimentally infected gerbils in tissue cysts. We performed a mass-spectrometric analysis of adult *P*. *kellicotti* soluble somatic protein (SSPs) extracts, excreted/secreted proteins (ESPs) produced by adult worms during *in vitro* culture, and lung cyst fluid proteins (CFPs) from experimentally infected gerbils. We identified 2,137 *P*. *kellicotti* proteins that were present in at least two of three biological replicates and supported by at least two peptides. Among those were 1,914 proteins found in SSP, 947 in ESP and 37 in CFP. *In silico* analysis predicted that only 141 of the total 2,137 proteins were secreted via classical or non-classical pathways. The most abundant functional categories in SSP were storage and oxidative metabolism. The most abundant categories in ESP were proteins related to metabolism and signal transduction. The 37 parasite-related proteins in CFP belonged to 11 functional categories. The largest groups were proteins with unknown function, cytoskeletal proteins and proteasome machinery. 29 of these 37 proteins were shared among all three sample types. To our knowledge, this is the first study that compares *in vitro* and *in vivo* ESP for any *Paragonimus* species. This study has provided new insights into ESPs of food-borne trematodes that are produced and released *in vivo*. Proteins released at the host-parasite interface may help the parasite evade host immunity and may represent new targets for novel treatments or diagnostic tests for paragonimiasis.

## Author summary

Neglected tropical diseases include food-borne trematode infections such as paragonimiasis that causes severe illness with important social and economic consequences. In this study, we compared excretory/secretory products (ESPs) produced by the North American lung fluke *Paragonimus kellicotti* that are released during *in vitro* culture with those that are released *in vivo* inside the lungs of experimentally infected animals. We identified a total of 2,137 *P*. *kellicotti* proteins. These included 1,914 proteins in soluble extracts of whole flukes, 947 proteins identified in ESPs released during *in vitro* culture, and 37 proteins identified in fluid recovered from lung cysts that surround adult flukes in experimentally infected gerbils. The most abundant functional protein categories varied by sample type. To our knowledge, this is the first study that has compared proteins released *in vitro* and *in vivo* by adult *Paragonimus* flukes. Proteins released in vivo may help the parasite evade host immunity. They may have practical value as novel drug targets or lead to improved diagnostic tests for paragonimiasis.

## Introduction

Lung flukes of the genus *Paragonimus* include about 30 species that are mainly found in Asia, but also Africa and the Americas causing an estimated 23 million human infections [1]. *Paragonimus kellicotti* is the cause of North American paragonimiasis, and can lead to severe pulmonary and/or extra-pulmonary diseases in carnivorous mammals including humans. Human infections are rare, but the number of people diagnosed with this parasite species has recently been increasing [2]. Humans become infected via consumption of raw or undercooked freshwater crayfish that contain the infective metacercariae. *P*. *kellicotti* is the only species endemic to the USA and a good model for other *Paragonimus* species because infectious metacercariae are readily available; a small rodent model has been established and its whole genome has been sequenced [3,4].

Previous studies of *Paragonimus* secretomes were carried out mainly to investigate the composition and/or immunological roles of selected molecules [5–7]. Despite the technical progress of mass spectrometry, few proteomic studies have been conducted for any *Paragonimus* species. The excretory/secretory proteins (ESP) released by parasites and presented at the parasite-host interface are frequently proposed to be useful targets for drugs and/or vaccines [8–10]. These molecules are actively released from the parasite gut, excretory pores and surface tegument. They include proteins, carbohydrates, lipids and RNAs that are likely to be critically important for parasite survival in the mammalian host [11]. Characterization of these worm components and their functions within the host could improve our understanding of parasite-host interactions, and this could lead to the discovery of new strategies to combat these parasitic infections.

*In vitro* culture of adult flukes is the most common strategy to collect ESP and the implicit assumption is that these products resemble the excretome and secretome of parasites *in vivo*. However, verification of the worm integrity and viability during culture is a key step to avoid contamination by components derived from dying parasites. Furthermore, parasites maintained *in vitro* face different environmental conditions than parasites *in vivo*. For example the host’s immune system is not present *in vitro*. Therefore, the assumption that parasites release the same ESPs *in vitro* and *in vivo* is likely to be incorrect. In the gerbil model, adult *P*. *kellicotti* flukes live individually or in small groups of two to four flukes in lung cysts. Analysis of fluid from these cysts offer the unique opportunity to study ESPs *in vivo* [3].

The aims of this study were to study ESPs to improve understanding of parasite-host interactions and to support the discovery of novel diagnostic or therapeutic targets. We used tandem mass-spectrometry to characterize the secretome of adult *P*. *kellicotti* flukes and to compare ESPs released during *in vitro* culture with parasite cyst fluid proteins (CFPs) which correspond to ESPs released by adult flukes *in vivo*. We also compared these results to the global proteome of soluble, somatic protein (SSP) extracts of whole adult flukes.

## Materials and methods

### Ethics statement

Mongolian gerbils (*Meriones unguiculatus*) aged 5 to 8 weeks old were purchased from Charles River Laboratories (Worcester, MA, USA) and kept under specific pathogen free conditions in an animal care facility of the Department of Comparative Medicine at Washington University in St. Louis. (Missouri, USA). A protocol for the ethical use of animals in research was approved by the university’s Institutional Animal Care and Use Committee (IACUC) (protocol ID 20–0503). Procedures involving animals were performed by specially trained personnel.

### Sample collection and preparation

*P*. *kellicotti* metacercariae were isolated from the heart tissue of two naturally infected species of crayfish (*Orconectes luteus* and *Orconectes punctimanus*) that were collected in Huzzah Creek (Missouri, USA) as previously described [3]. A total of four or five metacercariae, in 150μL phosphate buffered saline (PBS), were inoculated into each gerbil by intraperitoneal injection. Infected animals were sacrificed about 5 weeks post-infection, and necropsies were performed. Lungs were examined for cysts and adult flukes. Adult flukes recovered from cysts were washed three times for 5 min in warm sterile PBS and maintained in warm PBS until they emptied their gut contents. After that, parasites were incubated at 37°C, 5% CO_2_ in 500μL of sterile culture medium (RPMI 1640 supplemented with 30mM HEPES pH 7.2, 2% glucose and 10% penicillin/streptomycin/amphotericin mix; Sigma, St. Louis MO, USA). After 12hrs, the supernatant containing the ESP was collected under laminar flow hood. The fluid was syringe-filtered through a 0.22μm filter and store at -20°C until use.

For SSP, adult parasites (collected as described below) were washed three times for 5 min in warm and sterile PBS until they cleaned their gut content. Individual parasites were placed in a 1.5mL sterile tube containing PBS and mechanically crushed with a sterile plastic pestle under a laminar flow hood. The samples were centrifuged at 20.000X g for 10 minutes at 4°C and the supernatant with extracted proteins was syringe-filtered through a 0.22μm filter and transferred into a clean 1.5mL tube and store at -20°C until use.

Cyst fluid was collected by syringe aspiration of intact lung cysts from three different gerbils. The fluid was pooled and syringe-filtered through a 0.22μm filter and placed in a 1.5mL sterile tube and store at -20°C until use.

### Protein digestion and sample preparation

Peptides were prepared as previously described using a modification of the filter-aided sample preparation method [12]. Data were acquired as three technical replicates from single biological preparations for ESP, SSP and CFP samples types. Three aliquots of 15 μg total protein from each sample were mixed with 100 μl of 100 mM Tris-HCL buffer, pH 8.5 containing 8 M urea (UA buffer) and 10 mM DTT and reduced at room temperature for an hour. The samples were transferred to the top chamber of a 10,000 MWCO cutoff filtration unit (MilliporeSigma, Burlington MA, USA) and processed to peptides as previously described [12]. The peptides were dried in a Savant DNA 120 Speedvac concentrator (Thermo Fisher Scientific, Waltham MA, USA) for 15 min. The dried peptides were dissolved in 1% (vol/vol) TFA and desalted using two micro-tips (porous graphite carbon, BIOMEKNT3CAR) (Glygen, Columbia MD, USA) on a Biomek NX Beckman robot (Beckman Coulter, Brea CA, USA), as previously described [13]. The peptides were eluted with 60 μl of 60% (vol/vol) MeCN in 0.1% (vol/vol) TFA and dried in a Savant DNA 120 Speedvac concentrator (Thermo Scientific) after adding TFA to 5% (vol/vol). The peptides were dissolved in 20 μl of 1% (vol/vol) MeCN in water. An aliquot (10%) was removed for quantification using the Pierce Quantitative Fluorometric Peptide Assay kit (Thermo Scientific). The remaining peptides were transferred to autosampler vials (Sun-Sri,), dried and stored at -80°C.

### LC-MS/MS and data analysis

The peptides were separated using a nano-ELUTE chromatograph (Bruker Daltonics, Bremen, Germany) interfaced to a timsTOF Pro mass spectrometer (Bruker Daltonics) with a modified nano-electrospray source (Bruker Daltonics). The mass spectrometer was operated in Parallel accumulation-serial fragmentation (PASEF) mode [14]. The samples in 2 μL of 1% (vol/vol) FA were injected at a flow rate of 350 nL / min onto the column (75 μm i.d. × 25 cm Aurora Series) with a CSI emitter (Ionopticks, Melbourne Victoria, Australia). The column temperature was set at 50°C. The column was equilibrated using constant pressure (800 bar) with 8 column volumes of solvent A (0.1% (vol/vol) FA). Sample loading was performed at constant pressure (800 bar) in 2 μL of Solvent A.

The peptides were eluted using the following gradient of Solvent B (0.1% (vol/vol) FA/MeCN): solvent A containing 2% B was increased to 17% B over 60 min, to 25% B over 30 min, to 37% B over 10 min, to 80% B over 10 min and constant 80% B for 10 min. The MS1 and MS2 spectra were recorded from m/z 100 to 1700. Suitable precursor ions for PASEF-MS/MS were selected from TIMS-MS survey scans by a PASEF scheduling algorithm [14]. A polygon filter was applied to the m/z and ion mobility plane to select features most likely to be multiply charged peptide precursor ions. Quadrupole isolation width was set to 2 Th for m/z < 700 and 3 Th for m/z > 700. The collision energy was ramped stepwise as a function of increasing ion mobility: 52 eV for 0–19% of the ramp time; 47 eV from 19–38%; 42 eV from 38–57%; 37 eV from 57–76%; and 32 eV for the remainder. The TIMS elution voltage was calibrated linearly using the Agilent ESI-L Tuning Mix (m/z 622, 922, 1222).

Data from the mass spectrometer were converted to peak lists using DataAnalysis version 5.2 (Bruker Daltonics). The MS2 spectra with charges +2, +3 and +4 were analyzed using Mascot software version 2.5.1 (Matrix Science, London, UK) [15]. Mascot was set up to search against a custom parasite database (Paragonimus kellicotti, 12,850 entries) or host database (Meriones unguiculatus, 38,763 entries) assuming the digestion enzyme was trypsin with a maximum of 4 missed cleavages allowed [4,16]. The searches were performed with a fragment ion mass tolerance of 50 ppm and a parent ion tolerance of 25 ppm. Carbamidomethylation of cysteine was specified in Mascot as a fixed modification. Variable modifications included deamidation of asparagine and glutamine, pyro-glutamate formation from n-terminal glutamine, oxidation of methionine and acetylation of protein n-terminus.

Scaffold version 5.0.1 (Proteome Software Inc., Portland OR, USA) was used to validate MS/MS based peptide and protein identifications. Peptide identifications were accepted if they could be established at greater than 58.0% probability to achieve an FDR less than 1.0% by the Scaffold Local FDR algorithm. Protein identifications were accepted if they could be established at greater than 95.0% probability and contained at least 2 identified peptides. Protein probabilities were assigned by the Protein Prophet algorithm [17]. Proteins that contained similar peptides and could not be differentiated based on MS/MS analysis alone were grouped to satisfy the principles of parsimony. The mass spectrometry proteomics data were deposited in the ProteomeXchange Consortium (http://proteomecentral.proteomexchange.org) via the MassIVE partner repository with the dataset identifier MSV000089589.

### Protein functional annotation, classification and visualization

In addition to annotation with the *P*. *kellicotti* and *M*. *unguiculatus* databases [4], proteins were functionally classified using a software developed and provided by Dr. José. M. Ribeiro [18]. The functionally annotated proteins for each dataset and sample were manually curated and plotted on a hyperlinked Excel spreadsheet (S1 and S2 Tables) (Microsoft, Redmond WA, USA). NSAF (Normalized Spectral Abundance Factor) values were used to determine the proteins relative abundance. Mean NSAF values from at least two replicates were combined and then divided by the total NSAF for the respective sample. NSAF values were recorded in an Excel spreadsheet as a percentage of the total NSAF for respective samples, the results were plotted on pie charts according to protein functional classes. Principal component analysis (PCA) and scatter plot were conducted using GraphPad Prism version 9.1.3 for Windows (GraphPad Software, San Diego CA, USA). Protein sequences were examined for the presence of signal peptides using the program SignalP 6.0 (https://services.healthtech.dtu.dk/service.php?SignalP).

## Results and discussion

### Proteomic profiles of *P*. *kellicotti* samples

**Fig 1:**
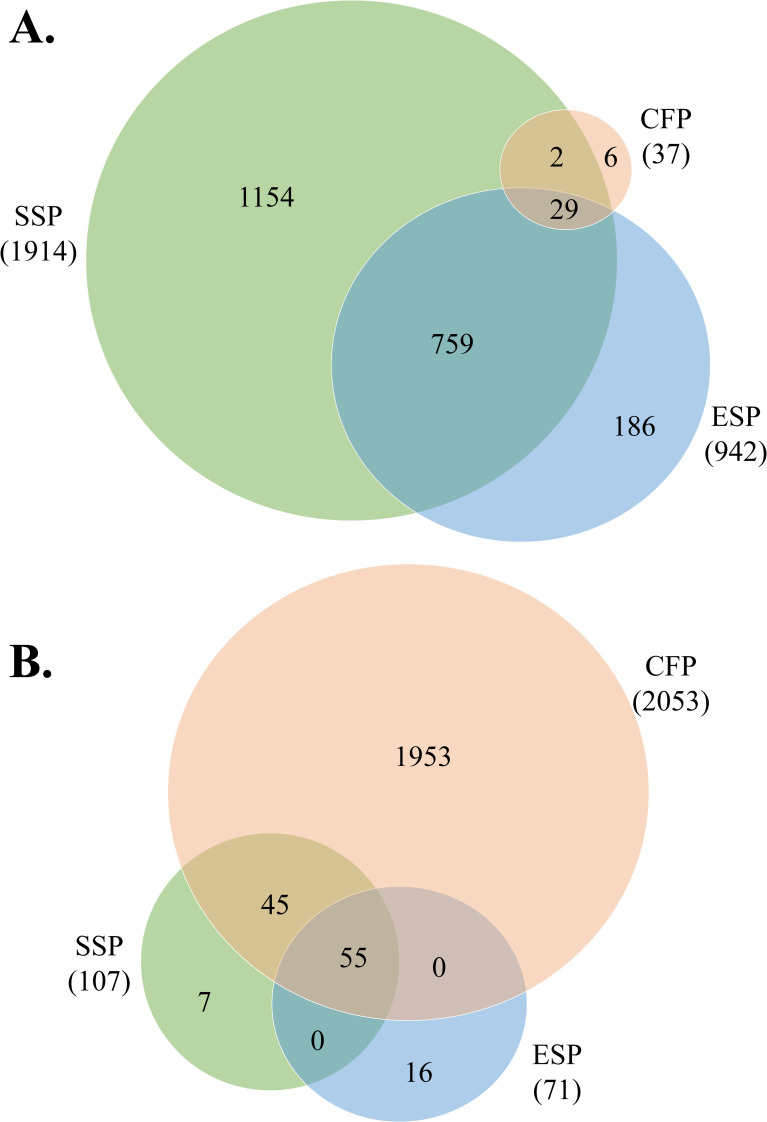
Venn diagrams representing the number of proteins found in each sample type, soluble somatic proteins (SSP), excretory/secretory proteins (ESP), cyst fluid proteins (CFP). The overlap region between the circles shows proteins present in two or more stages. In parenthesis is the total protein number for the sample. **A.** Comparison of *P*. *kellicotti*-derived proteins by sample type **B.** Comparison of *M*. *unguiculatus* (host)-derived proteins by sample type.

**Fig 2:**
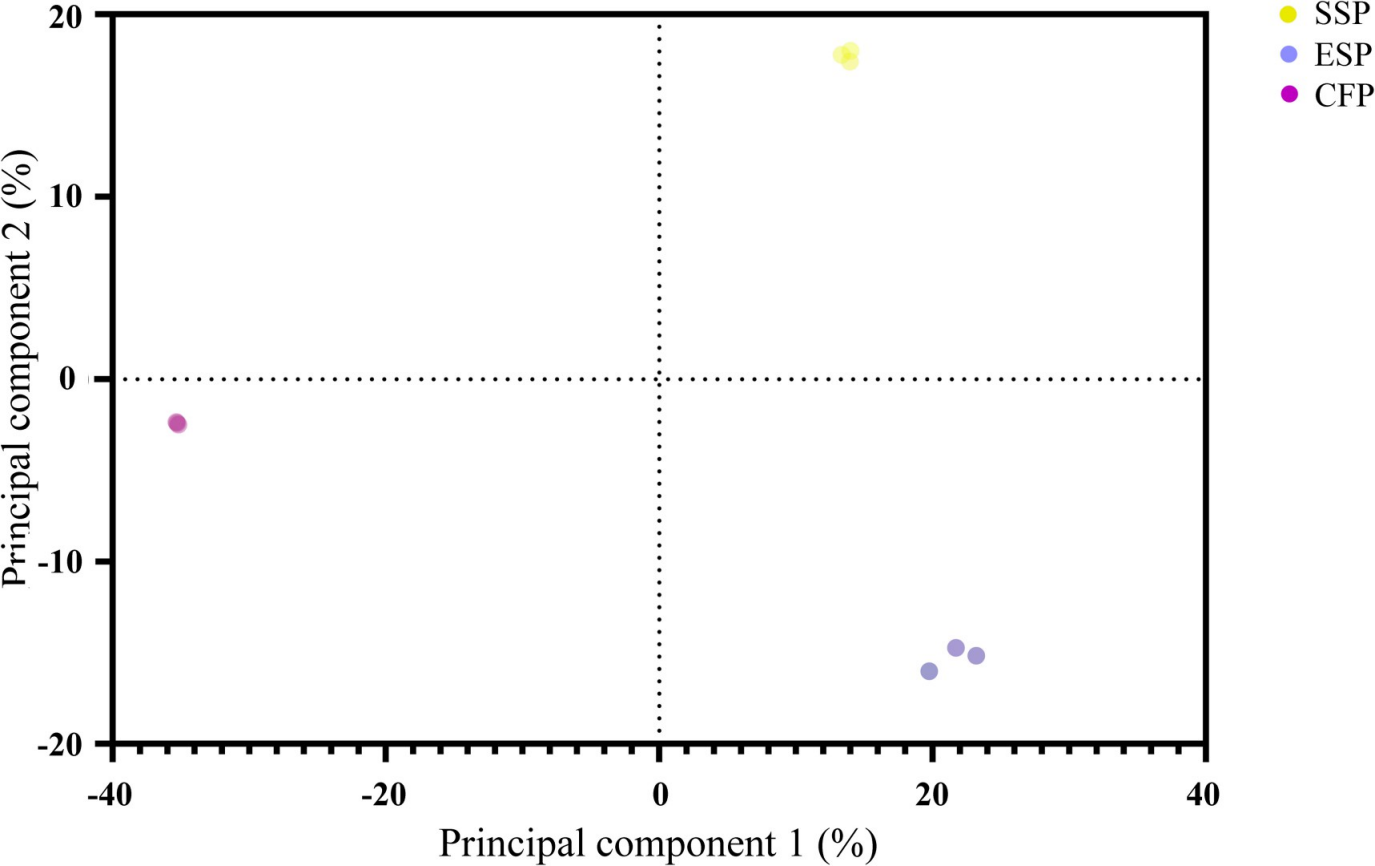
Principal component analysis. The PCA plot represents 2,137 parasite-related proteins with biological replicates that indicated distinct proteomics profile differences between soluble somatic proteins (SSP), excretory/secretory proteins (ESP), cyst fluid proteins (CFP) samples types represented with yellow, purple and pink dots, respectively.

A total of different 2,137 parasite-related proteins were identified and quantified using stringent criteria ≥ 2 peptides identified in at least 2 of the 3 replicate mass spectrometry runs from each sample (Fig 1A). ESP, SSP and CFP samples types were technically replicated in order to reduce the experimental noise of the results. Among these *P*. *kellicotti* proteins, 1,914 were detected in the SSP samples, 942 in ESP samples and 37 in CFP samples. The Venn diagram in Fig 1 shows that 29 *P*. *kellicotti* proteins were detected in all three sample types. Only 6 proteins were found exclusively in the CFP sample. These included 2 heat shock proteins, 1 histone H2A proteins and 2 proteins with unknown function (S1 Table). A principal component analysis (PCA) was applied to the protein expression data sets to show differences and similarities between sample types after functional classification of proteins (Fig 2). This analysis reduces the dimensionality of protein data sets to illustrate variation present in each data set [19]. This study is the second proteomic study of *P*. *kellicotti*. A previous mass spectrometry study to identify serodiagnostic targets by our group detected 2,555 predicted proteins in a single SSP sample [5]. While both studies used similar mass spectrometry instruments, the present study used an updated *P*. *kellicotti* database based on the recently published whole genome of *P*. *kellicotti* [4] and higher stringency analysis methods (e,g., use of NSAF values for quantitation).

*In silico* analysis predicted that only 141 (6.6%) of the total 2,137 proteins were secreted via a classical or non-classical pathway. These ESPs include 17 proteases, 16 metabolism related proteins, four transporters and three protease inhibitors (S1 Table). Among these 141 proteins, 112 were found in the SSP sample type (5.9% of the proteins detected in the sample), 50 in ESP sample type (5%) and only 1 CFP sample type (2.7%) (S1 Table). Proteins, including those that lack a signal peptide, can be also secreted or excreted by the intestine, excretory pores, surface cuticle/tegument shedding or release of extracellular vesicles (EV). The presence of EV may explain the detection of proteins that are assumed to have intra-trematode functions [20]. It was shown that EV can be taken up by host cells, a process that indicates a role in the parasite’s survival [21]. It was demonstrated that EVs differ in size, cargo and function depending on the developmental stages of the trematodes [22,23].

**Fig 3:**
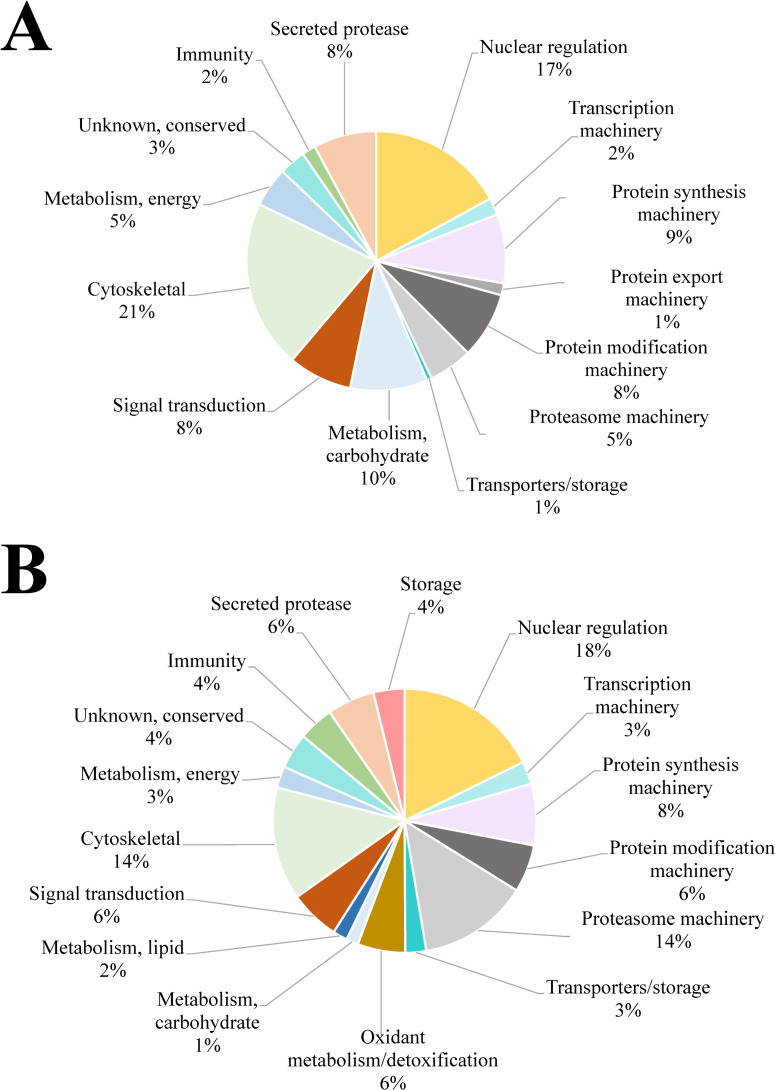
Functional annotation of *M*. *unguiculatus*-derived proteins found in *P*. *kellicotti* samples. Pie chart representing the percentage of proteins found in each sample with respect to NSAF values. **A**. soluble somatic proteins (SSP) sample type **B**. excretory/secretory proteins (ESP) sample type.

A total of 2,053 host-related proteins were detected in the CFP sample type and the most abundant proteins were plasma proteins and constitutive cell proteins (S3 Table). This is in accordance with the cyst architecture of the parasites, which consists of dense collagenous connective tissue and various inflammatory cells such as eosinophils. From the 128 host-derived proteins detected between the SSP and ESP samples types (Figs 1B and 3 and S2 Table); 55 proteins were shared between the three sample types and 7 and 16 host proteins were found exclusively in each sample, respectively. Based on relative abundance, most of the host proteins identified in the SSP and ESP samples type were blood or cytoskeletal proteins. Interestingly we were not able to find the most abundant blood proteins in SSP or ESP as was found in the CFP sample type (S3 Table). Thus, the presence of host-derived proteins in parasite secretions may be due to a recycling system and not due to contamination during sample collection. As it was described before, parasites were washed extensively and had no microscopically visible gut content before culture. Therefore, the only way to detect host proteins in the samples is if the parasites actively secrete them. For example in *F*. *hepatica* proteins of the host’s blood were found as part of the parasite EV content and in soft ticks as part of the salivary gland proteome [20,24,25]. Host proteins have been demonstrated in the *Schistosoma* tegument, and they may help the parasite to evade immune recognition [26].

### The somatic and secreted proteome of *P*. *kellicotti*

**Fig 4:**
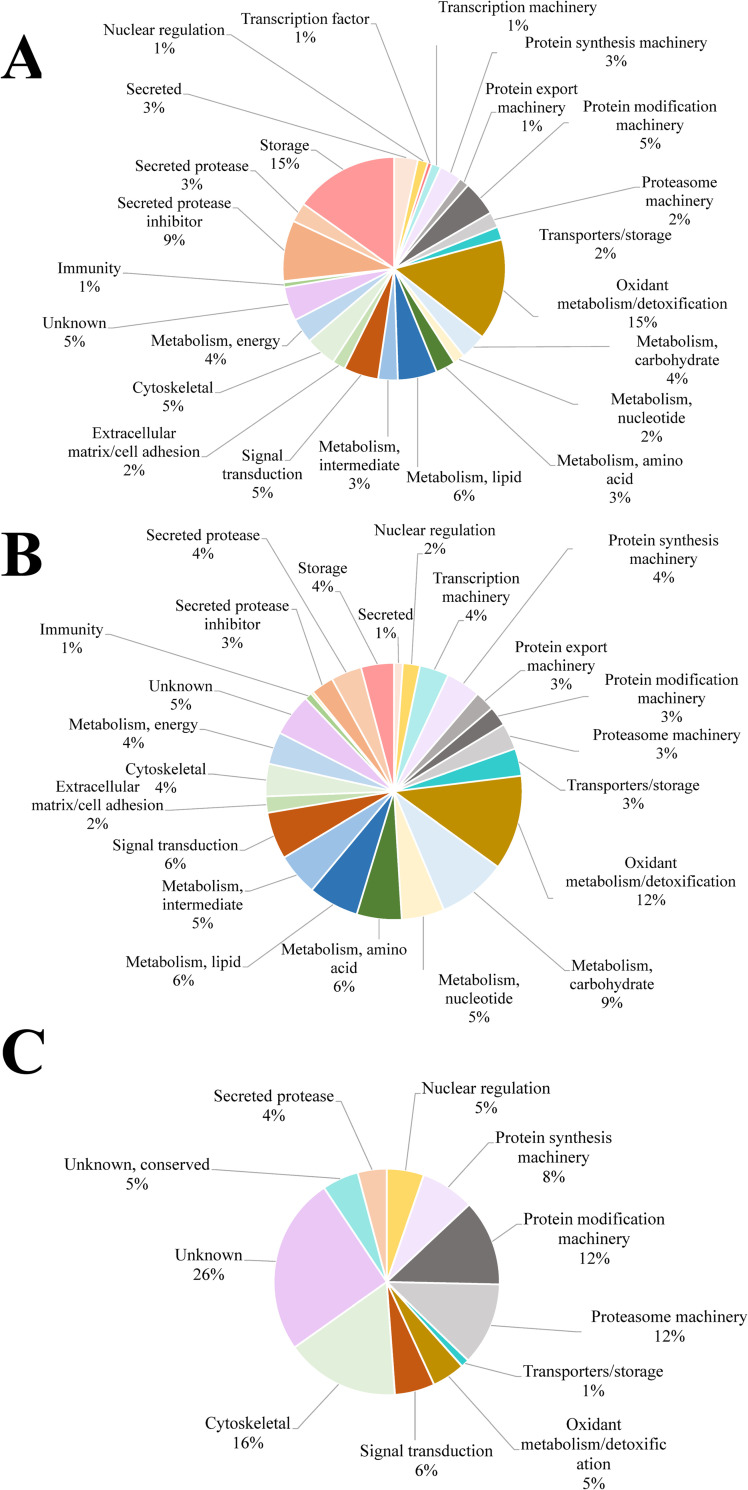
Functional annotation of *P*. *kellicotti*-derived proteins. Pie chart representing the percentage of proteins found in each sample type with respect to NSAF values. **A**. somatic soluble proteins (SSP) sample type **B**. excretory/secretory proteins (ESP) sample type **C.** cyst fluid proteins (CFP) sample type.

The most abundant functional categories in the SSP sample type were storage (15%), oxidant metabolism (15%) and protease inhibitors (9%) (Fig 4A). In contrast, the most abundant categories in the ESP sample type were metabolism related proteins (oxidation, carbohydrates and lipids, 12%, 9% and 6% respectively) and signal transduction (Fig 4B). The most abundant proteins identified in the SSP sample type were two glutathione S-transferases (GSTs), fatty acid-binding protein, calcium-binding protein and actin. This list overlapped significantly with the most abundant proteins in the ESP sample type (GSTs, ferritin, fatty acid-binding protein and two unknown proteins, see S1 Table).

GSTs are a versatile protein superfamily involved in cellular detoxification by either catalyzing toxin conjugation with glutathione (GSH) or passively binding to a wide range of endogenous/exogenous toxic molecules [27]. In parasites, GSTs participate in detoxifying exogenous toxins and protect the parasite from reactive oxidant damage as aldehyde products of lipid peroxidation in trematodes can be metabolized through glutathione conjugation [28]. Due to their important role in parasite-host interactions, GSTs have been targeted for pharmaceutical and vaccine purposes and have demonstrated protective effects against some parasites [29]. Fatty acid binding proteins (FABPs) are immunogenic proteins that play important roles in nutrient acquisition and parasite survival within the mammalian host. However as this family of proteins is often present in ESP, it may also have anti-inflammatory functions. For instance, a FABP secreted by *F*. *hepatica* induced human peripheral blood mononuclear cells (PBMCs) to express arginase, CHI3L1 and activated macrophages to release anti-inflammatory cytokines [30]. In addition, this FABP has been shown to downregulate production of nitric oxide and the expression of nitric oxide synthase. Several heat shock proteins (HSPs) were found in the SSP and ESP samples (17 and 8 proteins, respectively). HSPs are often detected in parasite ESPs, and they are major target of host immune responses [31]. HSPs function as proteins chaperones and they are believed to be important for stress resistance, parasite viability, infectivity and virulence [32].

Protease inhibitors play crucial roles in parasite development and survival; they are believed to counteract potentially damaging immune attack of vertebrate hosts. Many digenean trematodes rely on proteases to invade host tissues, and they also contribute to nutrition and development. We found 63 proteases in the SSP and 43 in the ESP sample with cysteine proteases as the most abundant subcategory. Several cysteine proteases were also found in *P*. *westermani* ESP, and some of them were recognized by antibodies in host serum samples [6]. Although we did not find many protease inhibitors (3%) in the *P*. *kellicotti* ESP samples, it is interesting that a cystatin was one of the most abundant proteins overall in the sample. Parasite cystatins not only participate in normal physiological processes, but they are also actively involved in host–parasite interactions including immune evasion [33]. The *P*. *kellicotti* cystatins share high sequence similarity with orthologs found in *S*. *japonicum* that are expressed in the gut and tegument of the adult parasite and inhibits the proteolytic activity of papain [34].

The study of the SSP and ESP can provide important clues to improve the serodiagnosis of parasitic diseases. For example, Sadaow and coworkers developed an immunochromatographic test for opisthorchiasis and clonorchiasis diagnosis using ESP [35]. The test used soluble ESP from adult worms cultured *in vitro* as the immobilized antigen. However, the preparation of ESP is time consuming and expensive, and it relies on the availability of adult parasites. In addition, ESP samples are difficult to standardize for use in serodiagnostic tests [36].

### Similarities and differences between proteins in ESP and CFP

The analysis of parasite ESP released *in vitro* provides a window into parasite-host interactions. However, ESP may also provide false signals. The presence of intracellular proteins or apoptotic factors in ESP suggest that some proteins detected are due to nonspecific leakage of intracellular proteins into the culture medium caused by cellular damage of the parasite [37]. *In vitro* culture conditions are different from those *in vivo* in mammalian hosts. Potential differences including pH, lack of exposure to the host’s immune system, nutrients, and host-regulated parasite density may account for some differences between *in vitro in vivo* ESPs. We identified 37 parasite-related proteins in CFP samples that could be divided in 11 functional categories (Table 1). The largest group consist of proteins with unknown function (without and with conserved domains, 31%), cytoskeletal proteins (16%) and proteasome machinery (12%) (Figs 1 and 4C). From these 37 proteins, 29 are shared among the three samples (Fig 4 and Table 1).

**Table 1:** Proteins found in the CFP sample type. In the first row are the accession numbers of each of the 37 parasite-derived proteins found in the CFP sample type. Presence of these proteins in the somatic soluble proteins (SSP) sample type and excretory/secretory proteins (ESP) sample type are marked with an asterisk. The GenBank accession number of the top blast hit and it similarity to the match is given in parenthesis. The proteins are ordered from the most to the least abundant in the CFP sample type.

CFP SAMPLE	SSP SAMPLE	ESP SAMPLE	DESCRIPTION	TOP BLAST HIT (ACCESSION NUMBER, %)
**KAF6774313.1**	*	*	Actin, alpha skeletal muscle	*Zea mays* (PWZ52530.1, 74)
**KAF6775699.1**			Unknown product	*Heterocephalus glaber* (EHB12217.1, 100)
**KAF6779974.1**	*	*	Heat shock protein 70	*Paragonimus skrjabini miyazakii* (KAF7255280.1, 96.2)
**KAF6775055.1**	*	*	Actin-1/4	*Schistosoma mansoni* (XP_018655545.1, 86.9)
**KAF6772180.1**	*	*	Polyubiquitin	*Ancylostoma duodenale* (KIH51178.1, 100)
**KAF6778982.1**			heat shock 70 kDa protein cognate 4	*Paragonimus westermani* (KAA3679877.1, 78.9)
**KAF6774896.1**	*	*	Transforming protein RhoA	*Paragonimus heterotremus* (KAF5401362.1, 97.4)
**KAF6775247.1**			heat shock protein 83-like	*Paragonimus westermani* (KAA3670344.1, 94)
**KAF6779118.1**	*	*	Elongation factor 2	*Paragonimus heterotremus* (KAF5400778.1, 95.2)
**KAF6775581.1**			Histone H2A	*Paragonimus westermani* (KAA3673838.1, 100)
**KAF6776868.1**	*	*	Ras-related protein Rab-7a	*Paragonimus westermani* (KAF8567099.1, 93.1)
**KAF6780400.1**	*	*	Calmodulin	*Corapipo altera* (XP_027493610.1, 98)
**KAF6775589.1**			Unknown product	*Opisthorchis viverrini* (XP_009166091.1, 100)
**KAF6768442.1**	*	*	ADP-ribosylation factor 1-like 2	*Paragonimus heterotremus* (KAF5397224.1, 86.7)
**KAF6777412.1**	*	*	Cathepsin F	*Paragonimus westermani* (KAA3681708.1, 55.7)
**KAF6772630.1**	*	*	Tubulin beta-4B chain	*Paragonimus skrjabini miyazakii* (KAF7258287.1, 100)
**KAF6776640.1**	*	*	Transitional endoplasmic reticulum ATPase	*Paragonimus skrjabini miyazakii* (KAF7256594.1, 99)
**KAF6778533.1**	*	*	Elongation factor 1-alpha	*Paragonimus skrjabini miyazakii* (KAF7255212.1, 94.7)
**KAF6768429.1**	*	*	Peroxiredoxin-2	*Paragonimus heterotremus* (KAF5399371.1, 96.3)
**KAF6777236.1**	*	*	Phosphoglycerate kinase	*Paragonimus skrjabini miyazakii* (KAF7232440.1, 97.3)
**KAF6776126.1**	*	*	Ras-related protein Rab-11B	*Paragonimus heterotremus* (KAF5402724.1, 97.2)
**KAF6776993.1**	*	*	Alpha-tubulin	*Chelonia mydas* (EMP31245.1, 85.6)
**KAF6780477.1**	*	*	Ras-related protein Rap-1b	*Paragonimus westermani* (KAA3675448.1, 99.4)
**KAF6780429.1**	*	*	Ras-related protein Rab-8B	*Paragonimus skrjabini miyazakii* (KAF7250710.1, 99.5)
**KAF6771111.1**	*	*	V-type proton ATPase subunit B	*Paragonimus heterotremus* (KAF5398304.1, 97.5)
**KAF6769383.1**	*	*	CPB2 protein	*Paragonimus pseudoheterotremus* (AOH96646.1, 76.8)
**KAF6779350.1**	*	*	T-complex protein 1 subunit delta	*Paragonimus heterotremus* (KAF5400171.1, 99)
**KAF6767940.1**	*		26S protease regulatory subunit 6B	*Paragonimus westermani* (KAA3677812.1, 100%)
**KAF6779213.1**	*		Spliceosome RNA helicase DDX39B	*Paragonimus westermani* (KAF8571704.1, 97.2)
**KAF6770190.1**	*	*	Tubulin beta-2A chain	*Macrostomum lignano* (PAA56989.1, 83.5)
**KAF6778686.1**	*	*	GTP-binding nuclear protein	*Paragonimus heterotremus* (KAF5399634.1, 77.7)
**KAF6768213.1**			AP complex subunit beta	*Paragonimus skrjabini miyazakii* (KAF7262109.1, 99.8)
**KAF6780438.1**	*	*	ATP synthase subunit alpha	*Paragonimus skrjabini miyazakii* (KAF7259739.1, 98.8)
**KAF6780309.1**	*	*	T-complex protein 1 subunit eta	*Paragonimus westermani* (KAA3675424.1, 98.3)
**KAF6779441.1**	*	*	Clathrin heavy chain	*Paragonimus westermani* (KAF8567126.1, 97.7)
**KAF6774619.1**	*	*	Sodium/potassium-transporting ATPase subunit alpha	*Paragonimus skrjabini miyazakii* (KAF7262433.1, 99.5)
**KAF6779958.1**	*	*	Spectrin beta chain	*Paragonimus heterotremus* (KAF5397619.1, 99.3)

Turning to parasite proteins shared by ESP and CSP, the most abundant of these proteins were peroxiredoxin-2, two cathepsins L, four Ras-related proteins and two T-complex protein 1 subunit. Peroxiredoxin are proteins able to play multiple physiological roles that include thiol-dependent peroxidase [38], chaperone holdase [39,40], regulator of H_2_O_2_, and modulator of the immune response [41,42]. Gene knock-out and RNA interference studies demonstrated that this protein plays a crucial role in survival and virulence, but there is evidences that its main physiological role may not be the same in different parasite species [43–45]. Parasite survival relies on an effective defense against oxidative damage due to reactive oxygen and nitrogen species produced by the host immune system. It was also demonstrated that peroxiredoxins could activate macrophages and play a key role in promoting parasite induced Th2 type immunity while suppressing a pro-inflammatory Th1 response. In parasitic trematodes, peroxiredoxins secreted in ESP inactivate reactive oxygen species, and act as pathogen-associated molecular pattern molecules (PAMPs) where they alternatively activate macrophages, using a mechanism that is independent of antioxidant function an suppress Th1 [41,46]. Cathepsin L is a cysteine protease related to papain. Although one of the two cathepsins found in these parasite-related shared proteins was annotated as a cathepsin F, after manually curated the database they were both reclassified as cathepsin L. The alignment with several well-known cathepsins L (S1 Fig) shows that they both have the ERFNIN, GNFD and GCNGG motifs (identified with an * on S1 Fig) characteristic of the cathepsin L. This enzyme plays several essential roles in parasite survival and host-parasite interactions. They participate in nutrient acquisition and they are part of the secretome of several parasites [47–49]. Cathepsin L have several functions such as nutrient acquisition by the digestion of host proteins, cleavage of the host extracellular matrix allowing parasite migration, and the impairment of the host immunity by destroying immunoglobulin and suppressing the Th1 immune response. One of the cathepsin (accession number KAF6769383.1) shares 70% sequence similarity with cathepsin Ls from *P*. *westermani* and *P*. *pseudoheterotremus* detected in adult SSP and ESP samples and is specifically expressed in the adult parasite digestive system [50]. They also demonstrate that this protein is highly sensitive and specific for serodiagnosis of human paragonimiasis, but no studies of species specificity or cross-reactivity have been performed. In *Fasciola gigantica*, cathepsin L was used as an antigen to develop a rapid diagnostic test for human *Fasciola* infection that was highly sensitive [51]. In the parasite-related shared proteins, four Ras-related proteins were found. Ras-related protein Rab-11B, Ras-related protein Rab-7a, Ras-related protein Rab-8B and Ras-related protein Rap-1b. These proteins belong to the small GTP-binding proteins family, part of the Ras superfamily, which regulate intracellular membrane trafficking of several parasites [52–54]. In *F*. *hepatica* was demonstrated that Ras proteins interacts with host immune system mediators and promotes monocytes phagocytosis and inhibits cell proliferation [52]. If most Ras-related proteins have a similar function when secreted, it may help explain how endoparasites recognize host immune cells and evade them to survive.

### Concluding remarks

To our knowledge, this is the first study that has compared *in vitro* and *in vivo* ESP for any *Paragonimus* species. Although analysis of ESPs produced *in vitro* can be helpful, in vitro culture does not reproduce the true biological and chemical conditions faced by the parasite inside the human host. Moreover, is well known that parasite secretomes undergo substantial changes in composition during their life cycle due to adaptation to different environments. Regarding the difference in the protein number between the ESP and CFP it is important to take into account that some proteins can be released into the cyst but diffuse from there to other host tissues. Therefore, the opportunity to analyze fluid from cysts that contained flukes allowed us to compare proteins secreted *in vivo* with proteins were found in culture medium after *in vitro* culture. Our results provide new insights into host-parasite interactions for an important food-borne parasitic trematode at the molecular level. Parasite proteins present in cyst fluid may also have practical value, because they could lead to new treatments or improved diagnostic tests for paragonimiasis.

## Supporting information

S1 Table Hyperlinked Excel spreadsheet with the functional annotation of the parasite-related-proteins and classification of the matched proteins by BLASTP searches against several databases for the three samples. (XLSX)

S2 Table Hyperlinked Excel spreadsheet with the functional annotation of the host-related-proteins and classification of the matched proteins by BLASTP searches against *M*. *unguiculatus* database for the SSP and ESP sample. (XLSX)

S3 Table Host derived protein list for the CFP. The proteins are ordered from the most to the least abundant in the sample.
(XLSX)

S1 Fig Amino acid sequences of *P*. *kellicotti* cathepsin L-like proteases (KAF6777412.1 and KAF6769383.1) aligned with known cathepsin L-like proteases retrieved from GenBank. Conserved cathepsin L motifs (ERFNIN, GNFD and GCNGG) were marked with an *.
(PDF)
