# Supplementary figures and images for: Comparative proteomics of adult Paragonimus kellicotti excretion/secretion products released in vitro or present in the lung cyst nodule
Source: PLoS Negl Trop Dis. 2022 Aug 17;16(8):e0010679. doi: 10.1371/journal.pntd.0010679 (PMC9423667; doi:10.1371/journal.pntd.0010679)

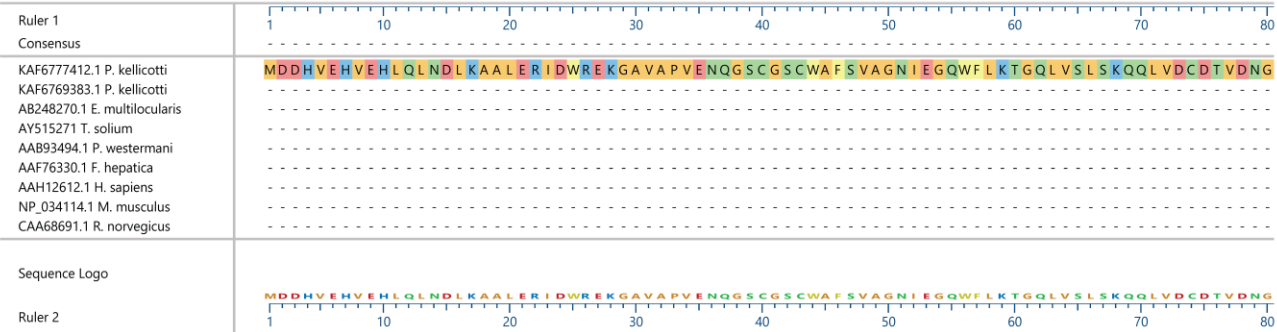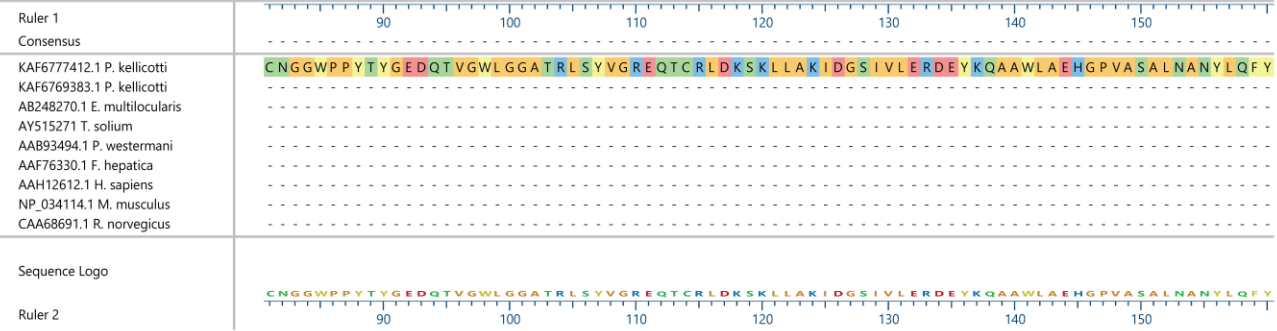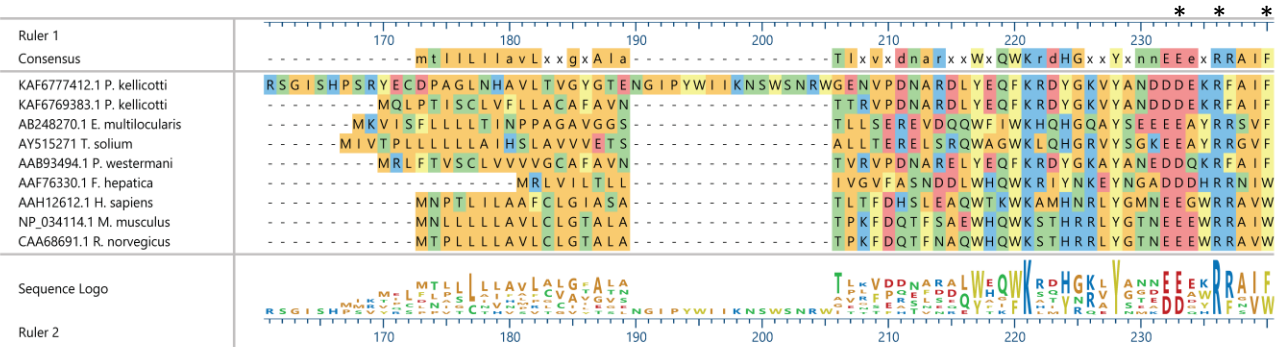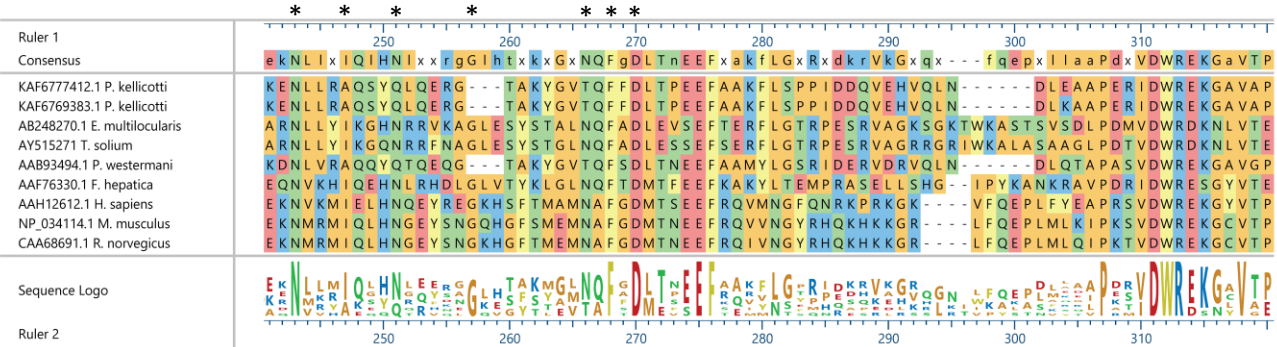

Supplement: S1 Fig — Conserved cathepsin L motifs (ERFNIN, GNFD and GCNGG) were marked with an *. (PDF) [file pntd.0010679.s004.pdf]
